# Segmental Glomerulosclerosis Subclassification in the Oxford Classification System (MEST-C) Improves the International IgA Nephropathy Prediction Tool

**DOI:** 10.3390/jcm15114036

**Published:** 2026-05-22

**Authors:** Yingting Du, Fang Lu, Zixuan Wang, Zihuan Qiu, Yifei Lu, Hua Shu, Yiyang Xu, Shan Hou, Zitao Wang, Bo Zhang, Changying Xing, Suyan Duan, Huijuan Mao, Yanggang Yuan

**Affiliations:** Department of Nephrology, the First Affiliated Hospital of Nanjing Medical University, Nanjing Medical University, 300 Guangzhou Road, Nanjing 210029, China; yingting_du@stu.njmu.edu.cn (Y.D.); njmulufang@stu.njmu.edu.cn (F.L.); wzx1124@stu.njmu.edu.cn (Z.W.); hz2025@stu.njmu.edu.cn (Z.Q.); yifeilu@stu.njmu.edu.cn (Y.L.); huashu@stu.njmu.edu.cn (H.S.); xyy02@stu.njmu.edu.cn (Y.X.); houshan1200@163.com (S.H.); xyadoc@njmu.edu.cn (Z.W.); zhangbo@jsph.org.cn (B.Z.); cyxing62@126.com (C.X.)

**Keywords:** IgA nephropathy, prognosis, international prediction tool, S subclassification, prediction model

## Abstract

**Background:** Early external validation studies demonstrated the robust and consistent predictive performance of the International IgA Nephropathy Prediction Tool (IIgAN-PT) across diverse ethnic populations. However, emerging evidence suggests that, in contemporary cohorts of patients with IgA nephropathy, the IIgAN-PT increasingly tends to overestimate the risk of adverse renal outcomes. Subclassification of segmental glomerulosclerosis (S lesions) in the Oxford Classification system (MEST-C) could identify high-risk IgAN patients, with evidence that different S subclassifications respond differently to treatment. Our study aimed to evaluate the predictive performance of the IIgAN-PT in a contemporary Chinese external validation cohort and to optimize its prognostic accuracy by incorporating the most severe and prevalent pathological subclassification of S lesions, NOS^+^Adh^+^, into the original model. **Methods:** A total of 746 Chinese patients were included with biopsy-proven IgAN in this study. Major adverse kidney events (MAKEs) were defined as death from any cause, initiation of renal replacement therapy, or a 50% decline in eGFR. This study evaluated the discrimination and model fit of three predictive models. The performance of the original and modified IIgAN-PT models was compared and evaluated through reclassification, survival analysis, calibration, decision curve analyses and subgroup analyses. **Results:** In the study cohort, the median follow-up duration was 4.2 years, during which 77 patients experienced MAKEs. The discriminative ability of the three original models was relatively limited. In contrast, the modified IIgAN-PT incorporating the NOS^+^Adh^+^ subtype of S subclassification demonstrated improved global performance for predicting 5-year risk, achieving a C-index of 0.808 (95% CI, 0.756–0.861). Kaplan–Meier survival curves showed clear risk stratification, particularly between low- and intermediate-risk categories. Reclassification analyses (continuous NRI and IDI) and decision curve analysis further supported enhanced predictive performance, while calibration curves corrected the original model’s risk overestimation. The modified model maintained stable performance across clinically relevant subgroups, including patients with hypertension, proteinuria, or receiving immunosuppression. **Conclusions:** This study further confirms the independent and clinically relevant prognostic value of the S pathological subclassification. The modified IIgAN-PT model, incorporating the NOS^+^Adh^+^ subtype of S subclassification, demonstrated consistent performance in individualized risk assessment for patients with IgA nephropathy.

## 1. Introduction

IgA nephropathy (IgAN) remains the most common primary glomerular disease [1,2], with approximately 30% to 45% of patients progressing to kidney failure within 20 to 25 years of diagnosis, particularly those with persistent proteinuria or unfavorable histological findings [3,4,5]. Early identification of patients at high risk of progression can support individualized clinical decision-making and improve long-term outcomes [6,7].

Several IgAN risk prediction models have been developed in recent years. These include (1) The International IgA Nephropathy Prediction Tool (IIgAN-PT), which combines the Oxford MEST-C pathologic score with clinical variables at biopsy and effectively predicts 5-year risk of renal failure [8]. The tool has also been extended for use at 1–2 years post-biopsy in adults and children [9]. It is recommended by the KDIGO 2025 Clinical Practice Guideline as a useful tool for assessing IgAN progression risk, although it does not address treatment response [10]. (2) The Kidney Failure Risk Prediction Equation (KFRE) was developed in a multicenter study based on a two-phase design. This study identified a best-performing model that incorporates clinical characteristics and the Oxford Score (CLIN-PATH) [11]. Finally, (3) The Simple Scoring Scale Model (SSM) uses the XGBoost algorithm to screen routinely available features and applies stepwise Cox regression to construct a restricted variable scoring model [12].

However, these models were developed using cohorts from earlier therapeutic eras, and their prediction windows do not extend to the median survival time of IgAN (approximately 10 years [3,13]). Moreover, none have been widely adopted in clinical practice. Recent clinical studies have substantially advanced IgAN management, leading to the adoption of SGLT2 inhibitors and the introduction of disease-modifying therapies such as targeted-release budesonide, dual endothelin-angiotensin receptor antagonists (e.g., sparsentan), and the complement inhibitor iptacopan [2,14,15,16,17]. These therapies are safe and effective, and combination therapy also shows promising results [18,19,20]. Consequently, these advances may compromise the predictive performance of the models described above. Therefore, reassessing the performance of existing prognostic models is essential to ensure their continued relevance and accuracy.

The VALIGA cohort showed that the segmental sclerosis (S) subclassification helps identify high-risk patients [21], our previous work also confirmed the prognostic utility of this subclassification in an Asian population [22]. Among S lesions, not otherwise specified (NOS) lesions with matrix expansion and capillary occlusion were the most common, followed by simple capsular adhesions (Adh) and podocyte hypertrophy (PH). Patients with NOS^+^Adh^+^ had heavier proteinuria, greater renal impairment, and worse outcomes, but also showed greater benefit from combined renin–angiotensin system blockade and immunosuppressive therapy [22]. These findings suggest that integrating the S subclassification into prediction models may improve their prognostic accuracy.

In this study, we retrospectively analyzed 746 IgAN patients treated at the First Affiliated Hospital of Nanjing Medical University. Our aims were: (1) to externally validate and compare risk prediction models for IgAN in a Chinese cohort; (2) to incorporate the most severe and prevalent NOS^+^Adh^+^ pathological subtype of S subclassification into the IIgAN-PT (Full Model With Race) to determine whether the modified model can better differentiate risk.

## 2. Materials and Methods

### 2.1. Study Design and Population

This retrospective cohort study included patients who underwent renal biopsy and were diagnosed with primary IgAN at the Department of Nephrology, The First Affiliated Hospital of Nanjing Medical University, from January 2014 to December 2019. IgAN was defined according to the KDIGO 2021 Clinical Practice Guideline [23]. Exclusion criteria were as follows: (1) eGFR < 15 mL/min/1.73 m^2^ or renal replacement therapy; (2) other systemic diseases involving the kidneys, such as diabetes mellitus, allergic purpura, vasculitis, and systemic lupus erythematosus; (3) incomplete baseline data; (4) <8 glomeruli in the renal biopsy specimen; (5) loss to follow-up.

The study was approved by the Ethics Committee of the First Affiliated Hospital of Nanjing Medical University (No.2024-SR-1037) and was conducted in accordance with the Declaration of Helsinki. All patients provided written informed consent. All participants were of Chinese ethnicity.

### 2.2. Clinical and Pathological Parameters

All clinical and pathological variables at renal biopsy and during follow-up were collected. These included age, sex, mean arterial pressure (MAP), serum creatinine (Scr), eGFR (calculated using the Chronic Kidney Disease Epidemiology Collaboration [CKD-EPI] equation [24]), 24 h urinary protein at biopsy, and use of renin–angiotensin–aldosterone system (RAAS) inhibitor and immunosuppression (IS). The use of RAAS inhibitor or immunosuppression was defined as any exposure at or before renal biopsy. Mean arterial pressure (MAP) was calculated as diastolic blood pressure plus one-third of pulse pressure. Renal biopsy specimens were evaluated using light microscopy, electron microscopy, and immunofluorescence staining techniques. Two expert pathologists independently graded the severity of kidney injury for each patient according to the Oxford MEST-C classification and performed subclassification of the S lesions. S1 was defined by the presence of either not otherwise specified (NOS) lesions and/or simple capsular adhesions. Specifically, we focused on the NOS^+^Adh^+^ subtype of S subclassification. To ensure the accuracy and reliability of the scoring, any discrepancies were reviewed through repeated discussion until a consensus was reached for every case [25].

### 2.3. Study Outcomes

The primary endpoint was major adverse kidney events (MAKEs), defined as death from any cause, initiation of renal replacement therapy, or a 50% decline in eGFR during follow-up, whichever occurred first. For patients who did not reach the endpoint, the end date was defined as the most recent hospitalization or clinic visit. Follow-up time for each patient was calculated from the date of hospital discharge after renal biopsy to the end date.

### 2.4. Statistical Analysis

Continuous variables are presented as mean ± standard deviation (SD) or median and interquartile range (IQR). Categorical variables are presented as counts and percentages. Three published risk prediction models, the International IgA Nephropathy Prediction Tool, the Kidney Failure Risk Equation, and the Simple Scoring Scale Model, were used to calculate the linear predictor (LP) and the predicted 5-year risk of major renal outcomes for each patient. Model discrimination was evaluated using the C-statistic. The Akaike information criterion (AIC) was used to assess overall model fit while accounting for model complexity, whereas the coefficient of determination (R^2^_D_) was used to estimate the proportion of variation explained in the outcome [26,27].

The modified IIgAN-PT model was developed based on the risk factors in the original IIgAN-PT model, with incorporation of the NOS^+^Adh^+^ subtype of S subclassification. The weight of each risk factor was derived from the adjusted hazard ratio (HR) estimated using the Cox proportional hazards regression model. Clinical risk reclassification was assessed using 5-year risk based on composite outcomes, including continuous net reclassification improvement (cNRI) and integrated discrimination improvement (IDI). Confidence intervals (CIs) were generated using internal validation with 1000 bootstrap samples. A 95% CI not including 0 for cNRI or IDI was considered statistically significant [8,28]. All evaluation metrics were derived using LP values.

Patients were categorized into four risk groups based on the percentile distribution of the linear predictor (LP) values: <16th percentile (low risk), ~16th to <50th percentile (intermediate risk), ~50th to <84th percentile (higher risk), and ≥84th percentile (highest risk). Kaplan–Meier curves were generated to estimate event-free survival for each risk group, and differences were assessed using the log-rank test [29]. Hazard ratios were calculated using the lowest-risk group as the reference to quantify the relative risk across groups. To assess calibration, histograms of predicted and observed 5-year risk probabilities were plotted for the four risk groups. Calibration plots were further generated by dividing patients into deciles of LP values, and decision curve analysis (DCA) was conducted to evaluate the models’ potential clinical utility. Additionally, subgroup ROC curves were constructed for proteinuria, hypertension, immunosuppression, eGFR, and other clinical subgroups. The performance of the original and modified models was evaluated separately within each subgroup [30].

All statistical analyses were performed using R statistical software (version 4.5.1). Statistical significance was defined as p < 0.05.

## 3. Results

### 3.1. Study Population Baseline Characteristics

Our cohort included 746 patients with IgAN, with a median follow-up of 4.2 years (IQR, 2.8–5.7). Baseline eGFR and median 24 h urinary protein were 102.1 mL/min/1.73 m^2^ and 0.8 g/d, respectively. A total of 81% of patients received a renin–angiotensin system (RAAS) inhibitor, and 53% were treated with immunosuppression (IS) after biopsy-confirmed diagnosis. Seventy-seven patients (10.3%) experienced a primary renal outcome, and 39 patients progressed to ESRD (5.2%). The clinical and pathological characteristics of the four study cohorts are summarized in Table 1. Compared with the other clinical cohorts, our cohort had a higher proportion of patients receiving immunosuppression (53.2% vs. 43.5% and 30.2%) and higher eGFR levels (102.1 vs. 83.0 and 74.7 mL/min/1.73 m^2^). Additionally, our cohort had lower E1, T1, and T2 scores in the MEST-C pathological system and demonstrated a better overall prognosis. The distribution of other clinical parameters, including age, sex, MAP, and urinary protein, was generally similar to that reported in the other three cohorts [8,11,12].

### 3.2. Measures of Discrimination and Model Fit

The linear prediction formulas and 5-year prediction probabilities for each reported model are listed in Table 2. Applied to our center’s IgAN cohort, the C-statistics from all three models did not exceed 0.80, indicating only moderate discriminative ability (IIgAN-PT: 0.775; CLIN-PATH: 0.792; SSM: 0.749). Furthermore, the R^2^_D_ values observed in our cohort were higher than those reported for the IIgAN-PT model (37.3% vs. 25.3%) and the CLINPATH model (36.6% vs. 20.5%) [8,11]. These findings suggest an improved capacity of the models to explain outcome variability in our external validation cohort (Table 3).

### 3.3. Performance of the Original and Modified IIgAN-PT

In this study, the most severe NOS^+^Adh^+^ (n = 388) subtype of S subclassification in the MEST-C pathological score was incorporated into both the IIgAN-PT and CLINPATH models. Both modified models demonstrated comparatively superior C-statistics. The C-index, R^2^_D_, and AIC values were similar between the two modified models, but all were better than those of the original versions (0.808 vs. 0.775 for IIgAN-PT, and 0.803 vs. 0.792 for CLINPATH). Notably, the improvement in C-statistic (ΔC) was greater for the modified IIgAN-PT model than for the modified CLINPATH model. For reclassification, compared with the SSM, the modified IIgAN-PT model showed greater improvement in both the continuous net reclassification index (cNRI) and integrated discrimination improvement (IDI). The values were 0.125 (95% CI, 0.000 to 0.230) for the cNRI and 0.044 (95% CI, −0.106 to 0.169) for the IDI. This enhancement was driven by the non-event group (0.163; 95% CI, 0.130 to 0.196), whereas reclassification performance in the event group showed a slight decline (−0.038; 95% CI, −0.151 to 0.058) (Table 4).

### 3.4. Comparison of Risk Groups

**Figure 1:**
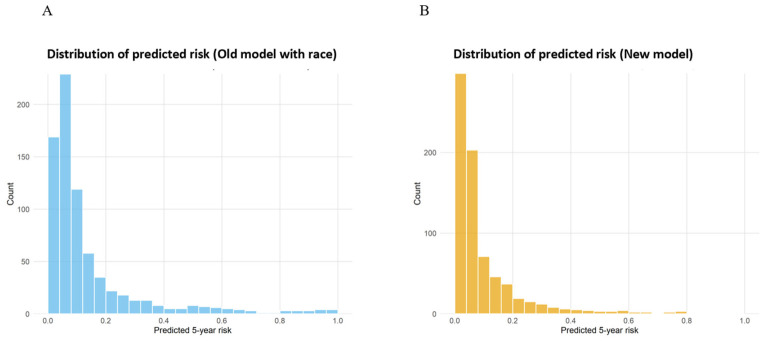
Histogram of the distribution in our cohort (n = 746) regarding the 5-year predicted risk of major adverse kidney events (MAKEs). Both models demonstrated a left-skewed predicted risk distribution, with the modified model assigning fewer patients to extreme high-risk categories. Count means frequency of IgAN patients. (**A**) Old model with race. (**B**) New model.

**Figure 2:**
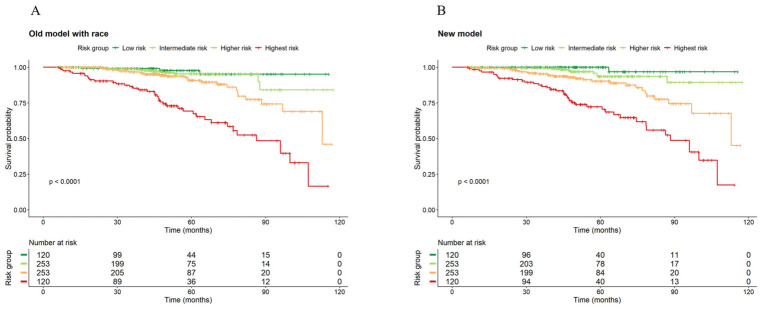
Kaplan–Meier curves of the primary outcome between the risk groups confirmed the better distinguishing ability of the new model, particularly evident between the low- and intermediate-risk groups. Risk groups were based on the percentiles of the linear predictor (low risk: <16th; intermediate risk: 16th–50th; higher risk: 50th–84th; highest risk: >84th). (**A**) Old model with race. (**B**) New model.

The frequency distributions of predicted risk for both the original and modified IIgAN-PT models were left-skewed (Figure 1), while the modified model classified fewer patients as being at extreme high risk compared with the original model. Patients were stratified into four risk groups according to LP percentiles, Kaplan–Meier curves, and hazard ratios between risk subgroups, which are presented in Figure 2 and Table 5. Compared with the original model, the modified IIgAN-PT incorporating the NOS^+^Adh^+^ subtype of S subclassification demonstrated superior risk discrimination on Kaplan–Meier survival curves. This improvement was particularly evident between the low- and intermediate-risk groups, while statistically significant differences were observed across the higher- and highest-risk groups. Although identical percentile-based grouping was applied in both models, recalculation of LP values in the revised model resulted in modest differences in the observed risks within each group.

### 3.5. Model Calibration

**Figure 3:**
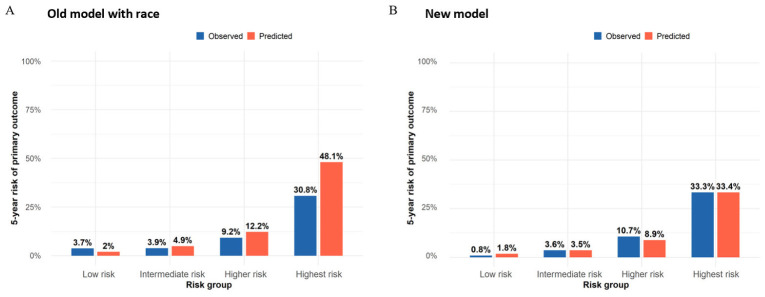
Comparison of observed and predicted 5-year risks of major adverse kidney events (MAKEs) in risk groups. The old model exhibited an overestimation of risk, particularly in the high-risk group, whereas the new model mitigated this overestimation. Risk groups were based on the percentiles of the linear predictor. (**A**) Old model with race. (**B**) New model.

**Figure 4:**
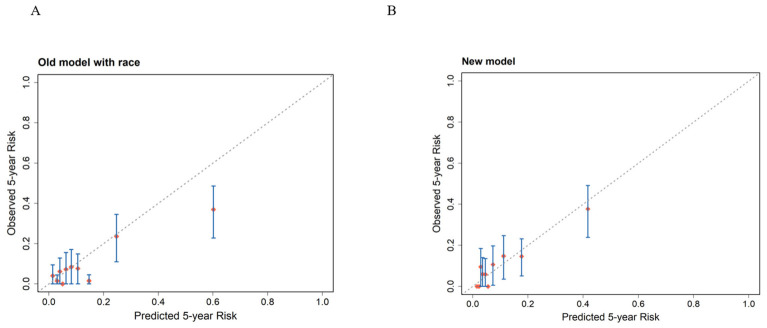
Calibration plots depict that the old model overestimated the risk of MAKEs, particularly in higher-risk deciles, whereas the new model achieved close agreement between predicted and observed risks, indicating improved calibration. The dashed lines indicate perfect calibration, in which predicted risks are exactly the same as the observed risks. Vertical lines (in blue) in observed groups represent 95% confidence intervals. Plots by tenths of predicted risk are in (**A**) the old model with race, and (**B**) the new model.

Adequate calibration is a fundamental requirement for any clinically useful prediction tool. In the original model, the 5-year predicted risks were 2.0%, 4.9%, 12.2%, and 48.1% across the four risk groups, with substantial overestimation in the highest-risk categories compared with the observed risks. After incorporating the NOS^+^Adh^+^ subtype of S subclassification, the modified model yielded 5-year predicted risks of 1.8%, 3.5%, 8.9%, and 33.4% across the four risk groups. This resulted in a marked improvement in the agreement between predicted and observed risks, particularly in the highest-risk group (Figure 3 and Table 5). Calibration curves stratified by risk group further confirmed that the modified model showed close concordance between predicted and observed 5-year risks (Figure 4).

### 3.6. Clinical Utility

**Figure 5:**
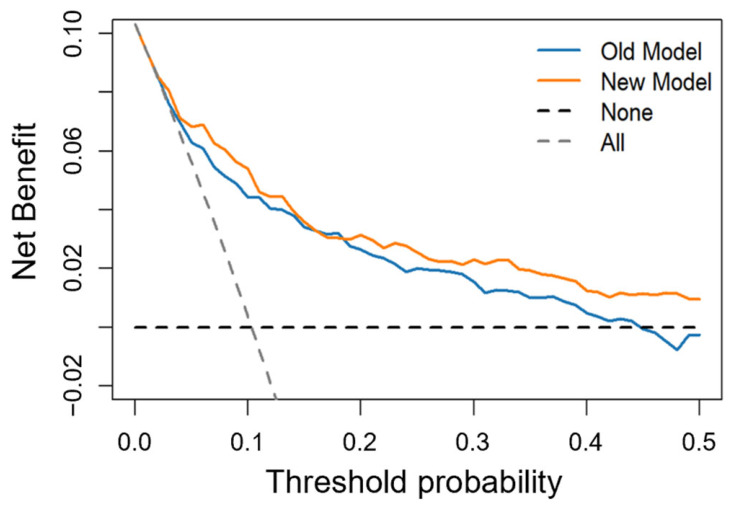
Decision curve analysis using 5-year predicted risk showed that the new model provided a modestly higher net benefit than the original model across clinically relevant risk thresholds, with occasional overlaps, and exceeded the net benefit of default strategies. The black dashed line represents the net benefit when no patients are considered as having the primary outcome; the gray dashed line represents net benefit when all patients are considered as having reached the outcome. The preferred model is the model with the higher net benefit at a reasonable range of probability thresholds.

Decision curve analysis (DCA) was used to assess whether the models could inform clinical decision-making (Figure 5). The new model demonstrated a positive net benefit across the entire range of 5-year predicted risk thresholds. Compared with the old model, the improvement was modest, and the curves overlapped at several thresholds. In contrast, the old model showed a negative net benefit within the 0.4–0.5 threshold range, indicating worse performance than a strategy that considers no patients at risk of the primary outcome.

### 3.7. Subgroup Analysis of the Original and Modified Model

**Figure 6:**
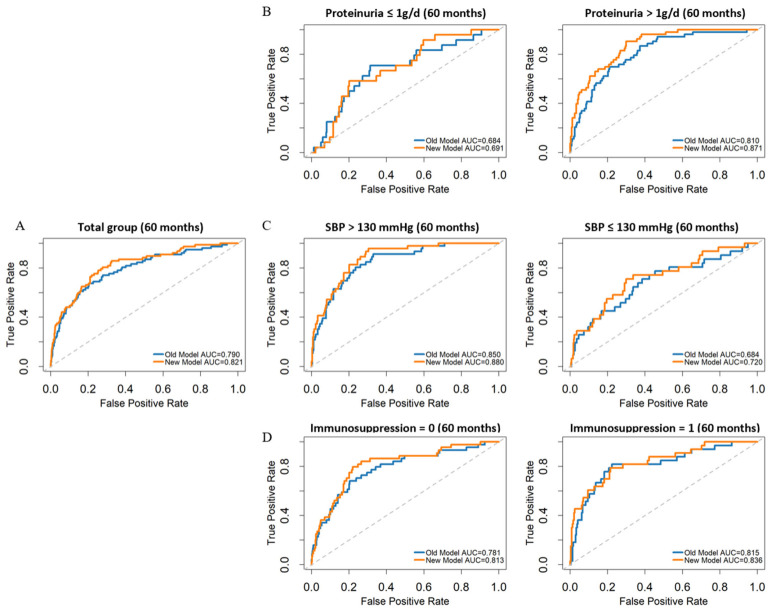
Receiver operating characteristic (ROC) curves of two models for predicting ESRD at 5 years in different subgroups. The modified model consistently outperformed the original model overall and across all subgroups. AUC, area under the receiver operating characteristic (ROC) curve, (**A**) in total patients; (**B**) in patients with baseline proteinuria ≤ 1 g/d and proteinuria > 1 g/d; (**C**) in patients with baseline SBP ≤ 130 mmHg and SBP > 130 mmHg; (**D**) in patients with or without immunosuppression.

Subgroup analyses were performed with stratification according to age, sex, systolic blood pressure, eGFR, proteinuria level, use of immunosuppression, and RAAS inhibition (Figure 6, Supplementary Figure S1). The modified model demonstrated superior 5-year predictive performance overall and across all subgroups. In the subgroups with proteinuria > 1 g/day or systolic blood pressure > 130 mmHg, both the modified and original models achieved AUCs greater than 0.8. This indicates good discriminatory performance for patients at relatively high risk. Notably, the AUC of the modified model remained above 0.8 regardless of immunosuppression use (Figure 6 and Table 6).

## 4. Discussion

In this study, we first externally validated three prediction models with complete risk formulas, including the International IgAN Prediction Tool (IIgAN-PT), the Kidney Failure Risk Prediction Equation (CLIN-PATH), and the Simple Scoring Scale Model (SSM). The discriminative performance of all three models was fair (C-statistic < 0.80), suggesting suboptimal model fit and the need for further refinement. Although the Oxford classification has been validated in large cohorts and provides a standardized and reliable framework for pathological assessment in IgA nephropathy, it does not capture all histopathological features with prognostic significance [31]. Additional histological characteristics may further enhance predictive value and improve the performance of the IIgAN-PT model. S1 was defined by the presence of either a NOS lesion and/or simple adhesions. Previous research from the Norwegian Kidney Biopsy Registry, which analyzed the S subclassification of segmental glomerulosclerosis (S) lesions, demonstrated that NOS lesions and perihilar glomerular sclerosis were associated with adverse renal outcomes [6]. Consistently, the large VALIGA cohort from 55 European centers identified NOS^+^Adh^+^PH^−^ or NOS^+^PH^+^ as being associated with higher proteinuria and poorer renal outcomes, while also showing a better response to immunosuppression [21]. Our prior research further validated the clinical relevance of these subtypes in Asian populations, although such subclassifications remain exploratory and are not included in the standardized Oxford classification system [22]. Therefore, we recalibrated the survival formula of the original IIgAN-PT model by replacing the S subclassification with the most severe NOS^+^Adh^+^ subtype.

The causes of the substantial clinical heterogeneity in IgA nephropathy (IgAN) remain unclear, but nearly all patients are at risk of developing kidney failure within their lifetime [32]. Therefore, early identification of prognostic risk factors and the development of prediction models are essential to guide timely detection and intervention in high-risk patients. The KDIGO 2025 Clinical Practice Guideline recommends the IIgAN-PT as a risk stratification tool, and multiple studies have validated its effectiveness and robustness across different ethnic groups [26,27,29,30,33]. However, these external validation cohorts were not prospective trial populations and did not inform treatment decisions. In recent years, advances in understanding the “four-hit hypothesis” have driven major progress in IgAN therapeutics, leading to the development and approval of several novel agents [32,34]. These developments highlight the need to reassess the applicability and accuracy of the IIgAN-PT model. For instance, a recent validation study of the updated IIgAN-PT model, involving patients with severe pathological features and widespread use of immunosuppression (IS), reported substantial overlap in survival curves between low- and intermediate-risk groups, with IS treatment significantly attenuating the model’s discriminative ability [35]. This aligns with findings from other contemporary real-world cohorts, in which the C-index of the IIgAN-PT model dropped from 0.82 to 0.74, accompanied by poor calibration and systematic risk overestimation [36]. In our cohort, the original IIgAN-PT (With Race) similarly overestimated the actual observed risk, whereas the modified model, incorporating the most severe NOS^+^Adh^+^ subtype, demonstrated improved predictive performance in terms of discrimination, calibration, reclassification, and clinical utility for predicting 5-year risk in our cohort. Survival curves stratified by LP percentiles showed good separation, potentially helping to avoid overtreatment and related toxicities in low-risk patients while focusing clinical resources on those at higher risk.

The modified IIgAN-PT model incorporating the NOS^+^Adh^+^ subclassification showed a modest increase in discrimination (C-index increase of 0.033, 95% CI: 0.020–0.047), and decision curve analysis indicated a similarly modest positive net benefit across the range of 5-year predicted risk thresholds. While the improvement reaches statistical significance, its practical impact on clinical decisions appears limited, and the clinical utility of the modified model warrants further investigation in external cohorts. Reclassification analyses further revealed that the modified model improved risk stratification mainly among non-event patients (NRI events = 0.163, 95% CI: 0.130 to 0.196), while slightly reducing the correct classification of patients who actually experienced adverse outcomes (NRI events = −0.038, 95% CI: −0.151 to 0.058). This indicates that, although overall performance is enhanced, the model remains limited in identifying high-risk patients. Future refinements, including consideration of immunosuppressive therapy subgroups or additional pathological features, may further improve prediction of adverse outcomes. Moreover, our model showed excellent predictive performance, with predicted probabilities in the highest-risk group differing from observed outcomes by only 0.1%. Nevertheless, the risk of overfitting cannot be excluded, which is potentially related to our optimization of all coefficients (including interaction terms), the use of center-specific pathology scoring, and the limitations inherent to a single-center, single-ethnicity cohort with a high rate of immunosuppression. Notably, more than half of our cohort received immunosuppression (IS), which has been reported in previous studies (e.g., VALIGA) to potentially modify the prognostic value of MEST-C components [21,31]. In our study, subgroup analyses by IS exposure showed that the model’s predictive performance was maintained and slightly improved in treated patients, indicating that IS did not weaken its utility. This improvement may result from immunosuppressive therapy reducing acute inflammatory activity, thereby allowing S lesions and clinical variables to more reliably reflect long-term renal risk. In addition, the full-race model exhibited discontinuities in risk prediction when applied to the Chinese population, potentially resulting in an overestimation of prognosis beyond 3 years [29]. Therefore, we prioritized further refinement of the race-inclusive model, with the aim of achieving improved predictive performance in the Chinese population. We also evaluated the IIgAN-PT model without race, in which the model demonstrated a relative underestimation of its predictive performance (Supplementary Figure S2).

This study has several strengths. First, the MEST-C histologic scoring system is relatively coarse and does not distinguish acute from chronic lesions [37,38], making the further subclassification of the segmental sclerosis (S) lesion a valuable refinement. Second, our cohort was established between 2014 and 2019, providing more recent biopsy data and a higher proportion of immunosuppression than earlier cohorts, thereby better reflecting contemporary IgAN management. However, several limitations should be noted. First, in the present study we only evaluated the impact of immunosuppression (as a binary variable) on model performance. With the widespread use of novel therapies for IgA nephropathy, such as SGLT2 inhibitors and targeted-release budesonide, further studies should determine whether incorporating more granular treatment-related variables improves predictive accuracy and consider whether to formally integrate these variables into the model. Second, given the single-center design, homogeneous ethnicity, and center-specific pathology scoring, the generalizability of the model and the robustness of the S lesion subclassification remain uncertain. In addition, the relatively low proportions of T1 and T2 lesions, together with the high rate of immunosuppression in our Chinese cohort, may limit the applicability of the findings. Therefore, extensive external validation in independent, multi-center, and multi-ethnic cohorts is critically needed to confirm the reproducibility of our results, evaluate the consistency of pathology scoring across centers, and assess potential overfitting. Such validation is essential before the model can be considered for broader clinical use. Third, the IIgAN-PT is applicable up to 7 years post-biopsy, whereas IgAN often presents in younger individuals and may progress over decades [3,39]. Long-term, stage-specific prognostic studies are needed to more accurately assess the benefits and risks of particular interventions and to identify clinically meaningful thresholds for initiating treatment [7].

## 5. Conclusions

In summary, our study developed a modified IIgAN-PT pathological model that incorporates the S subclassification, shows better overall performance, and provides more accurate prognostic risk estimation. However, further refinement of this model is needed to ensure its consistency with the emerging mechanism-based, stratified, and staged intervention paradigm for IgAN.

## Figures and Tables

**Table 1:** Characteristics of participants in the IIgAN-PT, CLINPATH, SSM and our validation cohorts at the time of kidney biopsy.

Characteristics	Our Validation Cohort	IIgAN-PT	CLINPATH	SSM
Patients, No.	746	2781	934	1022
Follow up, median (IQR), y	4.2 (2.8–5.7)	4.8 (3.0–7.6)	4.7 (1.0–25.0)	7.9 (6.6–9.8)
Age, y	38.9 (28.0–48.0)	35.6 (28.2–45.4)	36.5 ± 12.0	34.6 ± 9.4
Male, n (%)	341 (45.7)	1608 (57.8)	462 (49.5)	526 (48.5)
Scr, median (IQR), μmol/L	75.4 (60.9–101.5)	92.0 (70.7–123.8)	-	-
eGFR, mL/min/1.73 m^2^	102.1 (74–116.8)	83.0 (56.7–108.0)	74.7 ± 32.7	90.7 ± 29.2
<30, n (%)	27 (3.6)	142 (5.1)	-	-
30–60, n (%)	98 (13.1)	657 (23.6)	-	-
60–90, n (%)	150 (20.1)	800 (28.8)	-	-
≥90, n (%)	471 (63.1)	1182 (42.5)	-	-
MAP, mmHg	94 (82.3–104.9)	96.7 (88.7–106.3)	96.4 ± 12.7	96.0 ± 11.8
Proteinuria, g/d	0.8 (0.3–1.8)	1.2 (0.7–2.2)	1.1 (0.4–3.2)	1.0 (0.7–1.6)
<0.5, n (%)	276 (37.0)	383 (13.9)	-	-
∼0.5–1, n (%)	146 (19.6)	772 (28.1)	-	-
∼1–2, n (%)	142 (19.0)	817 (29.7)	-	-
∼2–3, n (%)	56 (7.5)	360 (13.1)	-	-
≥3, n (%)	104 (13.9)	415 (15.1)	-	-
MEST histologic score, n (%)				
M1	187 (25.1)	1054 (38.0)	233 (38.5)	262 (25.6)
E1	18 (2.4)	478 (17.3)	202 (33.3)	129 (12.6)
S1	597 (80.0)	2137 (77.0)	505 (83.3)	718 (70.3)
T1	75 (10.1)	686 (24.7)	152 (25.0)	194 (19.0)
T2	15 (2.0)	128 (4.6)	84 (13.8)	41 (4.0)
Crescents	359 (48.1)	953 (34.3)	-	-
RAASi, n (%)	602 (80.7)	2400 (86.7)	591 (73.0)	968 (94.7)
Immunosuppression, n (%)	397 (53.2)	1209 (43.5)	-	309 (30.2)
Primary outcome, n (%)	77 (10.3)	492 (17.7)		
50% decline in eGFR	38 (5.1)	420 (15.1)	-	
ESRD	39 (5.2)	372 (13.4)	132 (14.1)	

Note: Data are presented as median (Q1, Q3), mean ± standard deviation, or count (percentage). Abbreviations: Scr, serum creatine; eGFR, estimated glomerular filtration rate; MAP, mean arterial pressure; RAASi, Renin–Angiotensin–Aldosterone System Inhibitor; ESRD, end-stage renal disease; IIgAN-PT, the international IgA nephropathy prediction tool; CLINPATH, the best model from KFRE based on clinical characteristics and Oxford score; SSM, the simplified scoring scale model.

**Table 2:** Survival equations and linear predictors from three reported models.

Models	Equations
IIgAN-PT	LP = −0.351 × [sqrt(eGFR) − 8.8] − 0.0002 × (MAP − 97) − 0.093 × [log(proteinuria) − 0.09] + 0.006 × [(MAP × log(proteinuria)) − 8.73] + 0.155 × M1 − 0.131 × E1 + 0.097 × S1 + 0.607 × T1 + 1.189 × T2 + 0.109 × T1 × log(proteinuria) − 0.339 × T2 × log(proteinuria) − 0.016 × (age-38) + 0.818 × Chinese_race + 0.246 × RASB − 0.225 × immunosuppression Predicted risk (60 m) = 1 − 0.94176^exp[LP]^
CLIN-PATH	LP = −0.0323 × (Age − 37.3)] − [0.0567 × (eGFR − 72.5)] + [0.6351 × (M − 0.39)] + [0.7452 × (T − 0.53) Predicted risk (60 m) = 1 − 0.9725^exp[LP]^
SSM	LP = 1.234 × T1 + 1.901 × T2 + 1.004 × (Global sclerosis > 25%) + 0.561 × (Urine protein > 1 g/d) Predicted risk (60 m) = 1 − 0.985^exp[LP]^
IIgAN-PT Incorporated S subclassification	LP = −0.4982 × [sqrt(eGFR) − 8.8] − 0.005627 × (MAP − 97) − 0.4487 × [log(proteinuria) − 0.09] + 0.007088 × [(MAP × log(proteinuria)) − 8.73] − 0.02425 × M1 − 0.4667 × E1 + 0.1782×T1 + 1.2460×T2-0.3687 × T1 × log(proteinuria) − 1.1122 × T2 × log(proteinuria) − 0.01404 × (age − 38) − 0.2241 × RASB − 0.2268 × immunosuppression + 0.6080 × NOS^+^Adh^+^ Predicted risk (60 m) = 1 − 0.94384^exp[LP]^

Note: The age variable, as age in years at kidney biopsy; eGFR is as calculated using the CKD-EPI creatinine equation (in mL/min/1.73 m^2^). Abbreviations: CKD-EPI, Chronic Kidney Disease Epidemiology Collaboration; eGFR, estimated glomerular filtration rate; MEST, mesangial (M) endocapillary (E) hypercellularity segmental sclerosis (S) interstitial fibrosis/tubular atrophy (T); LP, linear predictor; NOS^+^Adh^+^, positive for lesions not otherwise specified (NOS) and simple capsular adhesions (Adh).

**Table 3:** Discrimination and model fit measures for different models in our validation cohort.

Models	C-Statistic	AIC	R^2^_D_, %
IIgAN-PT	0.775 (0.709–0.841)	802.9	37.3
CLINPATH	0.792 (0.733–0.852)	864.3	36.6
SSM	0.749 (0.672–0.827)	820.2	31.2
IIgAN-PT Incorporated S subclassification	0.808 (0.756–0.861)	799.2	43.3
CLINPATH Incorporated S subclassification	0.803 (0.744–0.861)	787.8	46.9

Note: Discrimination was assessed using the C-statistic; values in parentheses represent 95% confidence intervals. Model fit was assessed using AIC and R^2^_D_, with an increase in R^2^_D_ and reduction in AIC suggesting better model fit. Abbreviations: AIC, Akaike information criterion; R^2^_D_, coefficient of determination.

**Table 4:** Performance of models with or without S subclassification for predicting 5-year risk of composite kidney endpoint.

Variables	IIgAN-PT Incorporated S Subclassification	CLINPATH Incorporated S Subclassification
ΔC statistic	0.033 (0.020–0.047)	0.011 (0.009–0.011)
Continuous NRI	0.125 (0.000–0.230)	0.108 (−0.021–0.242)
NRI: events	−0.038 (−0.151–0.058)	0.347 (0.224–0.480)
NRI: nonevents	0.163 (0.130–0.196)	−0.240 (−0.272–−0.210)
IDI	0.044(−0.106–0.169)	0.006(−0.021–0.039)

Note: Reclassification using the IDI and the continuous NRI overall and in subgroups was based on experience of the primary outcome event. For the change (Δ) in C-statistic, NRI, and IDI, statistically a significant improvement is indicated by a 95% confidence interval (CI) that does not include 0. Abbreviations: NRI, net reclassification improvement; IDI, integrated discrimination improvement.

**Table 5:** Hazard ratios and rate of MAKEs in subgroups based on the linear predictor from external validation.

Risk Group	HR (95% CI)	*p*-Value	Mean Predicted 5-yr Risk, %	Mean Observed 5-yr Risk, %
Old model				
Low risk	Reference		2.0	3.7
Intermediate risk	1.59 (0.50–6.37)	0.44	4.9	3.9
Higher risk	3.66 (1.36–13.63)	0.008	12.2	9.2
Highest risk	13.07 (5.03–47.87)	<0.001	48.1	30.8
*p*-value for trend		<0.001		
New model				
Low risk	Reference		1.8	0.8
Intermediate risk	3.03 (0.70–28.26)	0.15	3.5	3.6
Higher risk	8.54 (2.25–76.28)	<0.001	8.9	10.7
Highest risk	24.90 (6.69–220.77)	<0.001	33.4	33.3
*p*-value for trend		<0.001		

Note: Risk groups were based on the percentiles of the linear predictor (low risk: <16th; intermediate risk: 16th to 50th; higher risk: 50th to 84th; highest risk: >84th). The low-risk group is the reference group. *p* values are for the hazard ratios in the subgroups. Abbreviations: MAKEs: major adverse kidney events; HR: hazard ratio; CI: confidence interval; yr: year.

**Table 6:** The area under the curve (AUC) and 95% CIs of two models for predicting ESRD at 5 years in different subgroups.

Subgroup	IIgAN-PT Model (Old Model)	IIgAN-PT Incorporated S Subclassification (New Model)
Total (N = 746)	0.790 (0.733–0.847)	0.821 (0.771–0.872)
SBP ≤ 130 mmHg	0.684 (0.577–0.791)	0.720 (0.622–0.819)
SBP > 130 mmHg	0.850 (0.794–0.905)	0.880 (0.834–0.927)
Proteinuria ≤ 1 g/d	0.684 (0.572–0.796)	0.691 (0.594–0.789)
Proteinuria > 1 g/d	0.810 (0.749–0.870)	0.871 (0.826–0.917)
Immunosuppression = 0	0.781 (0.704–0.857)	0.813 (0.744–0.882)
Immunosuppression = 1	0.815 (0.729–0.900)	0.836 (0.758–0.914)

Abbreviations: SBP, systolic blood pressure.

## Data Availability

All data generated during these studies are included in the text, figures, and tables of this article and electronic Supplementary Material. Further enquiries can be directed to the corresponding author.

## Supplementary Materials

The following supporting information can be downloaded at: https://www.mdpi.com/article/10.3390/jcm15114036/s1, Figure S1: Receiver operating characteristic (ROC) curves of two models for predicting ESRD at 5 years among the remaining different subgroups; Figure S2: Application of the IIgAN-PT model without race in an external Chinese cohort suggested a relative underestimation of its predictive performance.

## References

[1] Cheung C.K., Alexander S., Reich H.N., Selvaskandan H., Zhang H., Barratt J. (2025). The Pathogenesis of IgA Nephropathy and Implications for Treatment. Nat. Rev. Nephrol..

[2] Rovin B.H., Barratt J., Cook H.T., Noronha I.L., Reich H.N., Suzuki Y., Tang S.C.W., Trimarch H., Floege J. (2025). KDIGO 2025 Clinical Practice Guideline for the Management of Immunoglobulin a Nephropathy (IgAN) and Immunoglobulin a Vasculitis (IgAV). Kidney Int..

[3] Pitcher D., Braddon F., Hendry B., Mercer A., Osmaston K., Saleem M.A., Steenkamp R., Wong K., Turner A.N., Wang K. (2023). Long-Term Outcomes in IgA Nephropathy. Clin. J. Am. Soc. Nephrol. CJASN.

[4] Reich H.N., Troyanov S.A.A., Scholey J.W., Cattran D.C., Toronto Glomerulonephritis Registry (2007). Remission of Proteinuria Improves Prognosis in IgA Nephropathy. J. Am. Soc. Nephrol..

[5] Selvaskandan H., Barratt J., Cheung C.K. (2024). Novel Treatment Paradigms: Primary IgA Nephropathy. Kidney Int. Rep..

[6] Haaskjold Y.L., Lura N.G., Bjørneklett R., Bostad L.S., Knoop T., Bostad L. (2023). Long-Term Follow-up of IgA Nephropathy: Clinicopathological Features and Predictors of Outcomes. Clin. Kidney J..

[7] Rivedal M., Nordbø O.P., Haaskjold Y.L., Bjørneklett R., Knoop T., Eikrem Ø. (2025). Lifetime Progression of IgA Nephropathy: A Retrospective Cohort Study with Extended Long-Term Follow-Up. BMC Nephrol..

[8] Barbour S.J., Coppo R., Zhang H., Liu Z.-H., Suzuki Y., Matsuzaki K., Katafuchi R., Er L., Espino-Hernandez G., Kim S.J. (2019). Evaluating a New International Risk-Prediction Tool in IgA Nephropathy. JAMA Intern. Med..

[9] Barbour S.J., Coppo R., Zhang H., Liu Z.-H., Suzuki Y., Matsuzaki K., Er L., Reich H.N., Barratt J., Cattran D.C. (2022). Application of the International IgA Nephropathy Prediction Tool One or Two Years Post-Biopsy. Kidney Int..

[10] Floege J., Barratt J., Cook H.T., Noronha I.L., Reich H.N., Suzuki Y., Tang S.C.W., Trimarchi H., Balk E.M., Gordon C.E. (2025). Executive Summary of the KDIGO 2025 Clinical Practice Guideline for the Management of Immunoglobulin a Nephropathy (IgAN) and Immunoglobulin a Vasculitis (IgAV). Kidney Int..

[11] Xie J., Lv J., Wang W., Li G., Liu Z., Chen H., Xu F., Sun J., Ouyang Y., Zhang X. (2018). Kidney Failure Risk Prediction Equations in IgA Nephropathy: A Multicenter Risk Assessment Study in Chinese Patients. Am. J. Kidney Dis..

[12] Chen T., Li X., Li Y., Xia E., Qin Y., Liang S., Xu F., Liang D., Zeng C., Liu Z. (2019). Prediction and Risk Stratification of Kidney Outcomes in IgA Nephropathy. Am. J. Kidney Dis..

[13] Shen X., Chen P., Liu M., Liu L., Shi S., Zhou X., Lv J., Zhang H. (2024). Long-Term Outcomes of IgA Nephropathy in China. Nephrol. Dial. Transplant..

[14] Barratt J., Rovin B., Diva U., Mercer A., Komers R. (2019). Implementing the Kidney Health Initiative Surrogate Efficacy Endpoint in Patients with IgA Nephropathy (the PROTECT Trial). Kidney Int. Rep..

[15] Lafayette R., Kristensen J., Stone A., Floege J., Tesař V., Trimarchi H., Zhang H., Eren N., Paliege A., Reich H.N. (2023). Efficacy and Safety of a Targeted-Release Formulation of Budesonide in Patients with Primary IgA Nephropathy (NefIgArd): 2-Year Results from a Randomised Phase 3 Trial. Lancet.

[16] Jongs N., Greene T., Chertow G.M., McMurray J.J.V., Langkilde A.M., Correa-Rotter R., Rossing P., Sjöström C.D., Stefansson B.V., Toto R.D. (2021). Effect of Dapagliflozin on Urinary Albumin Excretion in Patients with Chronic Kidney Disease with and without Type 2 Diabetes: A Prespecified Analysis from the DAPA-CKD Trial. Lancet Diabetes Endocrinol..

[17] Perkovic V., Barratt J., Rovin B., Kashihara N., Maes B., Zhang H., Trimarchi H., Kollins D., Papachristofi O., Jacinto-Sanders S. (2025). Alternative Complement Pathway Inhibition with Iptacopan in IgA Nephropathy. N. Engl. J. Med..

[18] Schanz M., Seikrit C., Hohenstein B., Meyer A.K., Kraft L., Schricker S., Schwab A., Oberacker T., Latus J. (2025). First Long-Term Real-World Evidence of Sparsentan Efficacy in Patients with IgAN Treated with SGLT2 Inhibitors: SA-PO0809. J. Am. Soc. Nephrol..

[19] Ling X., Cheng M., Ao Ieong C.W., Yichi Z., Ming Y., Shi X., Ning Z., Han S., Yang X. (2025). Beyond Nine Months: Real-World Efficacy and Safety of Extended Nefecon Therapy in IgAN: SA-PO0824. J. Am. Soc. Nephrol..

[20] Heerspink H.J.L., Kiyosue A., Wheeler D.C., Lin M., Wijkmark E., Carlson G., Mercier A.-K., Åstrand M., Ueckert S., Greasley P.J. (2023). Zibotentan in Combination with Dapagliflozin Compared with Dapagliflozin in Patients with Chronic Kidney Disease (ZENITH-CKD): A Multicentre, Randomised, Active-Controlled, Phase 2b, Clinical Trial. Lancet.

[21] Bellur S.S., Troyanov S., Vorobyeva O., Coppo R., Roberts I.S.D., Validation in IgA Nephropathy Study Group (2024). Evidence from the Large VALIGA Cohort Validates the Subclassification of Focal Segmental Glomerulosclerosis in IgA Nephropathy. Kidney Int..

[22] Lu F., Hou S., Liu C., Li Q., Wu L., Pan Y., Wu Y., Shu H., Zhang B., Mao H. (2025). Clinical Significance of Focal Segmental Glomerulosclerosis Subclassification in IgA Nephropathy. Nephrol. Dial. Transplant..

[23] Rovin B.H., Adler S.G., Barratt J., Bridoux F., Burdge K.A., Chan T.M., Cook H.T., Fervenza F.C., Gibson K.L., Glassock R.J. (2021). KDIGO 2021 Clinical Practice Guideline for the Management of Glomerular Diseases. Kidney Int..

[24] Levey A.S., Stevens L.A., Schmid C.H., Zhang Y.L., Castro A.F., Feldman H.I., Kusek J.W., Eggers P., Van Lente F., Greene T. (2009). A New Equation to Estimate Glomerular Filtration Rate. Ann. Intern. Med..

[25] Trimarchi H., Barratt J., Cattran D.C., Cook H.T., Coppo R., Haas M., Liu Z.-H., Roberts I.S.D., Yuzawa Y., Zhang H. (2017). Oxford Classification of IgA Nephropathy 2016: An Update from the IgA Nephropathy Classification Working Group. Kidney Int..

[26] Zhang Y., Guo L., Wang Z., Wang J., Er L., Barbour S.J., Trimarchi H., Lv J., Zhang H. (2020). External Validation of International Risk-Prediction Models of IgA Nephropathy in an Asian-Caucasian Cohort. Kidney Int. Rep..

[27] Zhang J., Huang B., Liu Z., Wang X., Xie M., Guo R., Wang Y., Yu D., Wang P., Zhu Y. (2020). External Validation of the International IgA Nephropathy Prediction Tool. Clin. J. Am. Soc. Nephrol..

[28] Guo Y., Ren Y., Shi S., Wang S., Zhou X., Liu L., Lv J., Zhu L., Zhang H. (2025). Effects of Podocyte Foot Process Effacement on Kidney Prognosis and Response to Immunosuppressive Therapy in IgA Nephropathy. Kidney Med..

[29] Bon G., Jullien P., Masson I., Sauron C., Dinic M., Claisse G., Pelaez A., Thibaudin D., Mohey H., Alamartine E. (2023). Validation of the International IgA Nephropathy Prediction Tool in a French Cohort beyond 10 Years after Diagnosis. Nephrol. Dial. Transplant..

[30] Ouyang Y., Zhao Z., Li G., Luo H., Xu F., Shao L., Chen Z., Yu S., Jin Y., Xu J. (2021). A Validation Study Comparing Risk Prediction Models of IgA Nephropathy. Front. Immunol..

[31] Coppo R., Troyanov S., Bellur S., Cattran D., Cook H.T., Feehally J., Roberts I.S.D., Morando L., Camilla R., Tesar V. (2014). Validation of the Oxford Classification of IgA Nephropathy in Cohorts with Different Presentations and Treatments. Kidney Int..

[32] Barratt J., Lafayette R.A., Floege J. (2024). Therapy of IgA Nephropathy: Time for a Paradigm Change. Front. Med..

[33] Hu L., Fang Y., Huang J., Liu J., Xu L., He W. (2024). External Validation of the International Prognosis Prediction Model of IgA Nephropathy. Ren. Fail..

[34] Perše M., Večerić-Haler Ž. (2019). The Role of IgA in the Pathogenesis of IgA Nephropathy. Int. J. Mol. Sci..

[35] Du X., Zhou J., Ma M., Yu H., Lv J., Lu X. (2025). External Validation of the Updated International IgA Nephropathy Prediction Tool in a Chinese Population. Eur. J. Med. Res..

[36] Shen X., Chen P., Liu L., Shi S., Zhou X., Barbour S.J., Lv J., Zhang H. (2025). Novel Therapies Improve Prognosis of IgAN and Limit the Applicability of the International IgA Nephropathy Prediction Tool. Clin. Kidney J..

[37] Kang D., Ban T.H., Chin H.J., Lee H., Oh S.W., Park C.W., Yang C.W., Choi B.S. (2022). Prognostic Value of Chronicity Grading on Renal Outcomes in Patients with IgA Nephropathy. Front. Med..

[38] Howie A.J., Lalayiannis A.D. (2023). Systematic Review of the Oxford Classification of IgA Nephropathy: Reproducibility and Prognostic Value. Kidney360.

[39] Knoop T., Vågane A.M., Vikse B.E., Svarstad E., Magnúsdóttir B.T., Leh S., Varberg Reisæter A., Bjørneklett R. (2015). Addition of eGFR and Age Improves the Prognostic Absolute Renal Risk-Model in 1,134 Norwegian Patients with IgA Nephropathy. Am. J. Nephrol..

