# Supplementary material for: Segmental Glomerulosclerosis Subclassification in the Oxford Classification System (MEST-C) Improves the International IgA Nephropathy Prediction Tool
Source: J Clin Med. 2026 May 22;15(11):4036. doi: 10.3390/jcm15114036 (PMC13257388; doi:10.3390/jcm15114036)
Supplement: Supplementary file 1 [file jcm-15-04036-s001.zip › jcm-4283055-supplementary.pdf]

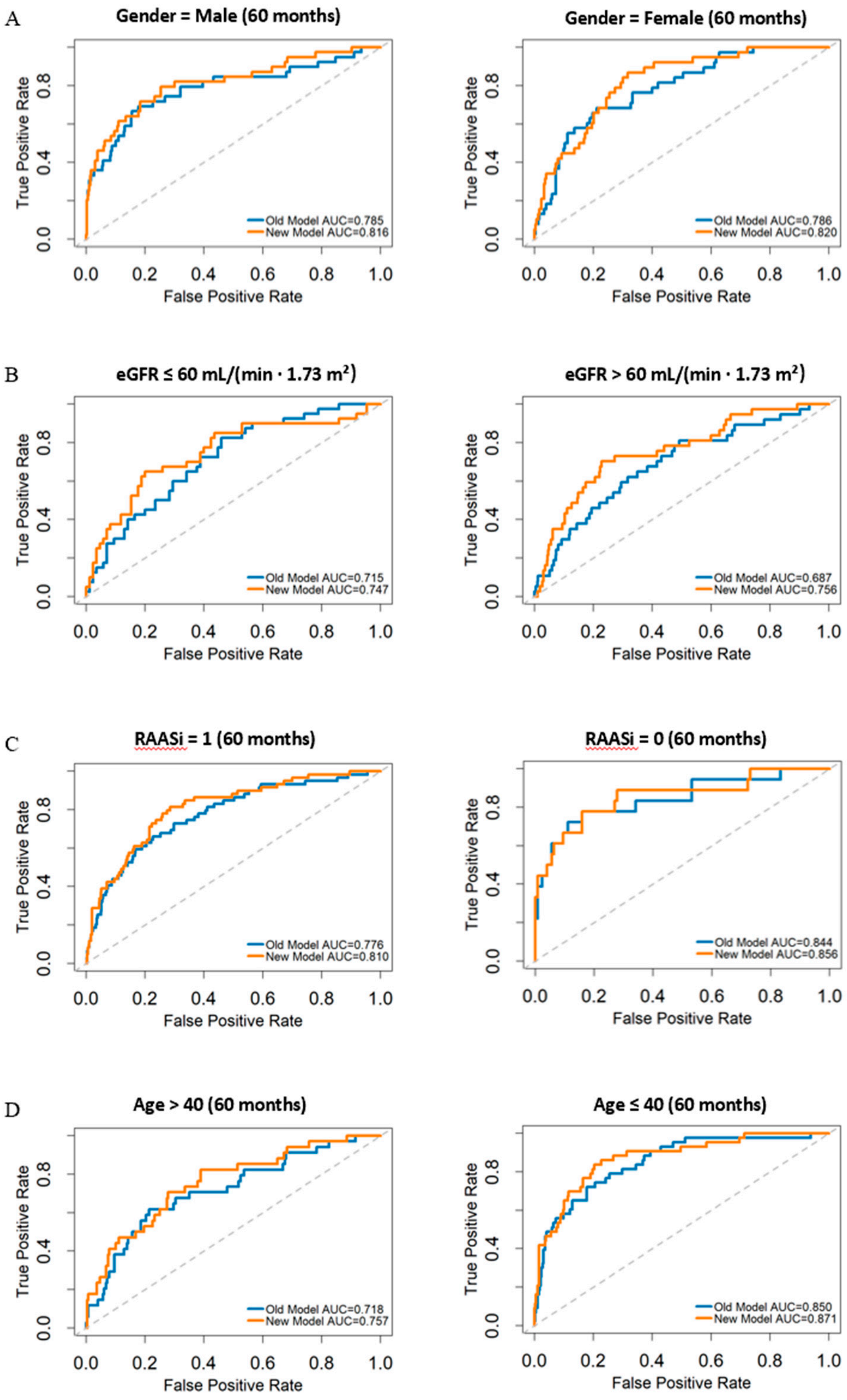

Supplemental Figure S1. Receiver operating characteristic (ROC) curves of two models for predicting ESRD at 5 years among the remaining different subgroups. The modified model consistently outperformed the original models. AUC, area under the receiver operating characteristic (ROC) curve. (A) in the subgroup of gender; (B) in the subgroup of eGFR; (C) in the subgroup of RAAS inhibitors; (D) in the subgroup of age.

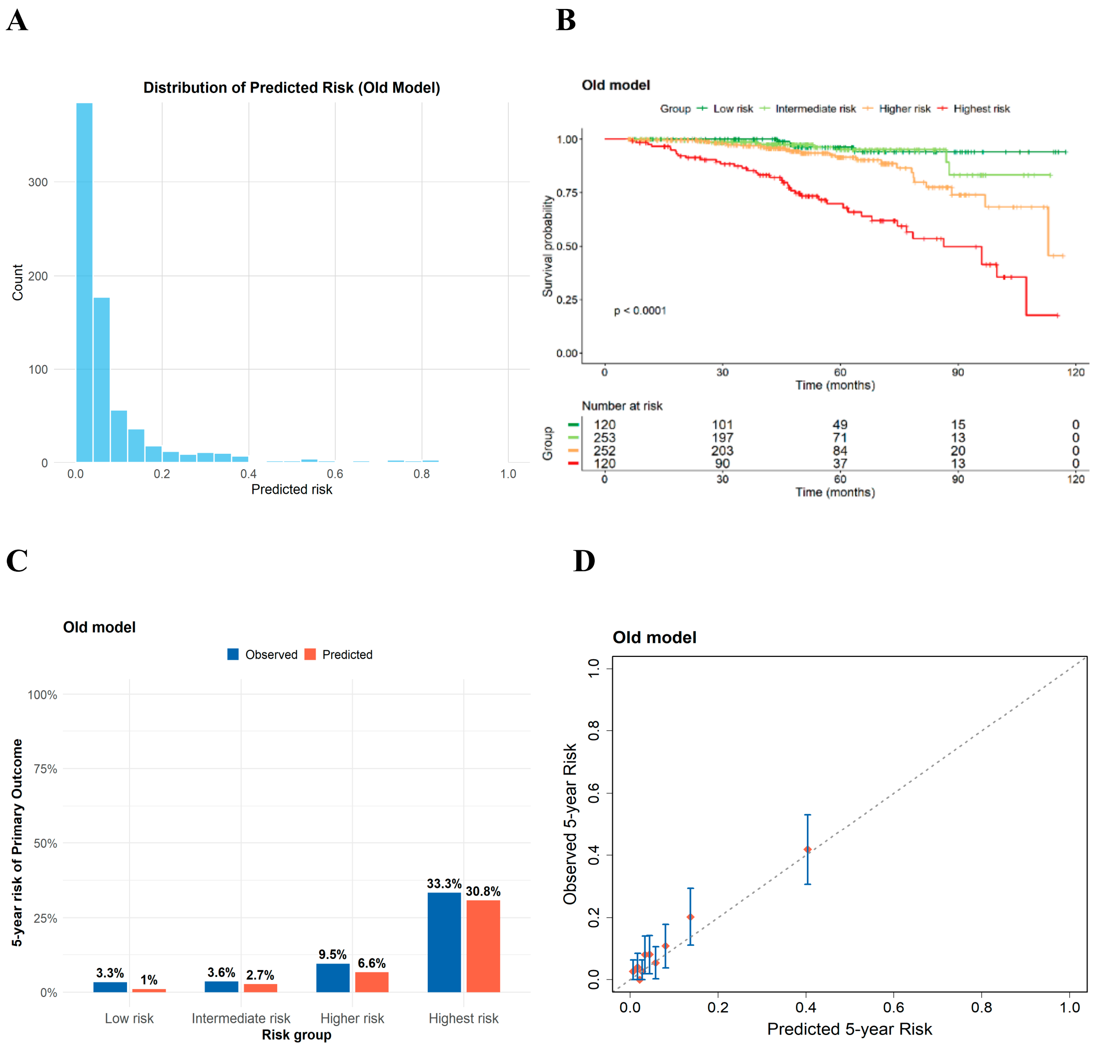

Supplemental Figure S2. Application of the IIgAN-PT model without race in an external Chinese cohort suggested a relative underestimation of its predictive performance. (A) Histogram of the distribution in our cohort (n=746) of the major adverse kidney events (MAKE). (B) Kaplan–Meier curves between the risk groups (C) Comparison of observed and predicted 5-year risks in risk groups. (D) Calibration plots for predicting 5-year risk.
